# The Effects of Selective Laser Trabeculoplasty in Patients With Angle Recession Glaucoma: A Case Series

**DOI:** 10.7759/cureus.73749

**Published:** 2024-11-15

**Authors:** Jason Dossantos, Sinan Akosman, Julie Thomasian, Devin Hill, Shelly Mishra, Stephen Lesche, David Belyea

**Affiliations:** 1 Department of Ophthalmology, George Washington University School of Medicine and Health Sciences, Washington, D.C., USA; 2 Department of Ophthalmology, Brooke Army Medical Center, San Antonio, USA

**Keywords:** angle recession glaucoma, glaucoma practice, glaucoma therapy, primary open angle glaucoma, selective laser trabeculoplasty

## Abstract

Purpose: To evaluate the effectiveness of selective laser trabeculoplasty (SLT) in reducing intraocular pressure (IOP) and medication use in treated and untreated eyes of angle recession glaucoma (ARG) patients within a year post procedure.

Methods: A retrospective chart review was conducted on nine ARG patients treated with SLT at George Washington University between January 1, 2008, and January 1, 2022. Patients were excluded if they had no ARG diagnosis, did not undergo SLT, lacked follow-up within one year after SLT, or had undergone laser or glaucoma surgery in the treated or untreated eye within 12 months before SLT. Primary outcomes were IOP reduction and SLT success, defined as a ≥20% reduction in IOP without additional IOP-lowering procedures or medications. Follow-up assessments were conducted at six weeks, six months, and 12 months. Statistical analysis included paired t-tests and ANOVA.

Results: A total of nine eyes from nine ARG patients were included, with a mean age of 62.33 years. SLT treatment success was observed in five out of nine (55.5%) treated eyes at various time points. In untreated contralateral eyes, success was noted in three out of nine patients (33.3%). The mean IOP for SLT-treated eyes at baseline, six-week, six-month, and 12-month visits was 20 ± 6.22 mmHg, 17.39 ± 5.11 mmHg (P > 0.05), 18.69 ± 4.99 mmHg (P > 0.05), and 16.83 ± 3.87 mmHg (P > 0.05), respectively. For the same time points, the mean IOP for untreated eyes was 15 ± 3.28 mmHg (P > 0.05), 14 ± 3.20 mmHg (P > 0.05), 14.38 ± 4.24 mmHg (P > 0.05), and 14.83 ± 3.82 mmHg (P > 0.05), respectively. The average number of medications used for the ipsilateral eye at the baseline, six-week, six-month, and 12-month visits was 3.11 ± 1.27, 3.11 ± 1.05, 3.25 ± 1.04, and 3.50 ± 0.55, with no significant changes over time (P > 0.05). Meanwhile, for the contralateral eye, the average medication use at those same points was 1.00 ± 1.32, 1.00 ± 1.32, 1.13 ± 1.36, and 0.67 ± 0.82, also showing no significant changes (P > 0.05).

Conclusion: SLT was safe and effective in reducing IOP in a subset of treated ARG eyes, with better outcomes observed in those with higher baseline IOP. While individual responses varied, SLT could be considered a viable treatment option for delaying invasive surgery in ARG patients. Larger studies are needed to confirm long-term efficacy and identify predictors of success.

## Introduction

Angle recession glaucoma (ARG) is a subtype of open-angle glaucoma (OAG) that often occurs secondary to ocular trauma. This condition is hypothesized to arise from the disruption of trabecular meshwork and ciliary body, resulting in elevated intraocular pressure (IOP) and potential glaucomatous optic neuropathy [1,2]. A rare condition, ARG was found to develop in only 6% of eyes with angle recession over 10 years in a prospective study by Kaufman and Tolpin [1]. The progression to glaucoma is influenced by various factors, including the extent of angle recession, persistent elevation of IOP, poor initial visual acuity, advancing age, and the presence of hyphema or lens injury [3-6]. Notably, one study found that 50% of ARG patients may develop OAG in the contralateral eye, suggesting that angle recession can accelerate glaucoma progression in predisposed eyes rather than directly cause elevated IOP [7].

Traditional surgeries like trabeculectomy have been reported as effective treatments for ARG patients; however, the efficacy of laser trabeculoplasty in this group remains unclear [8]. A study by AlObaida et al. found that selective laser trabeculoplasty (SLT) was successful in reducing IOP in three out of four eyes during their follow-up period [9]. Conversely, Robin et al. reported that argon laser trabeculoplasty (ALT) did not achieve treatment success in any of the four eyes treated [10]. Grouping both SLT and ALT together, an analysis of the Intelligent Research in Sight (IRIS®) Registry suggests that patients with ARG have a 1.69-fold increased likelihood of inadequate IOP reduction post-laser trabeculoplasty [11].

Given the small sample size in prior studies and the lack of differentiation between ALT and SLT in the IRIS® analysis, further investigation into the effect of SLT in ARG patients is warranted to better understand its potential benefits and limitations. Additionally, examining the effects of SLT in the untreated contralateral eye is particularly important given the frequent occurrence of contralateral OAG in ARG patients, especially since prior studies on the contralateral effect have not specifically investigated this in the context of ARG [12]. This study aims to investigate the IOP-lowering effect of SLT in the treated and untreated eyes of ARG patients within one year following the procedure.

## Materials and methods

Study design

**Figure 1:**
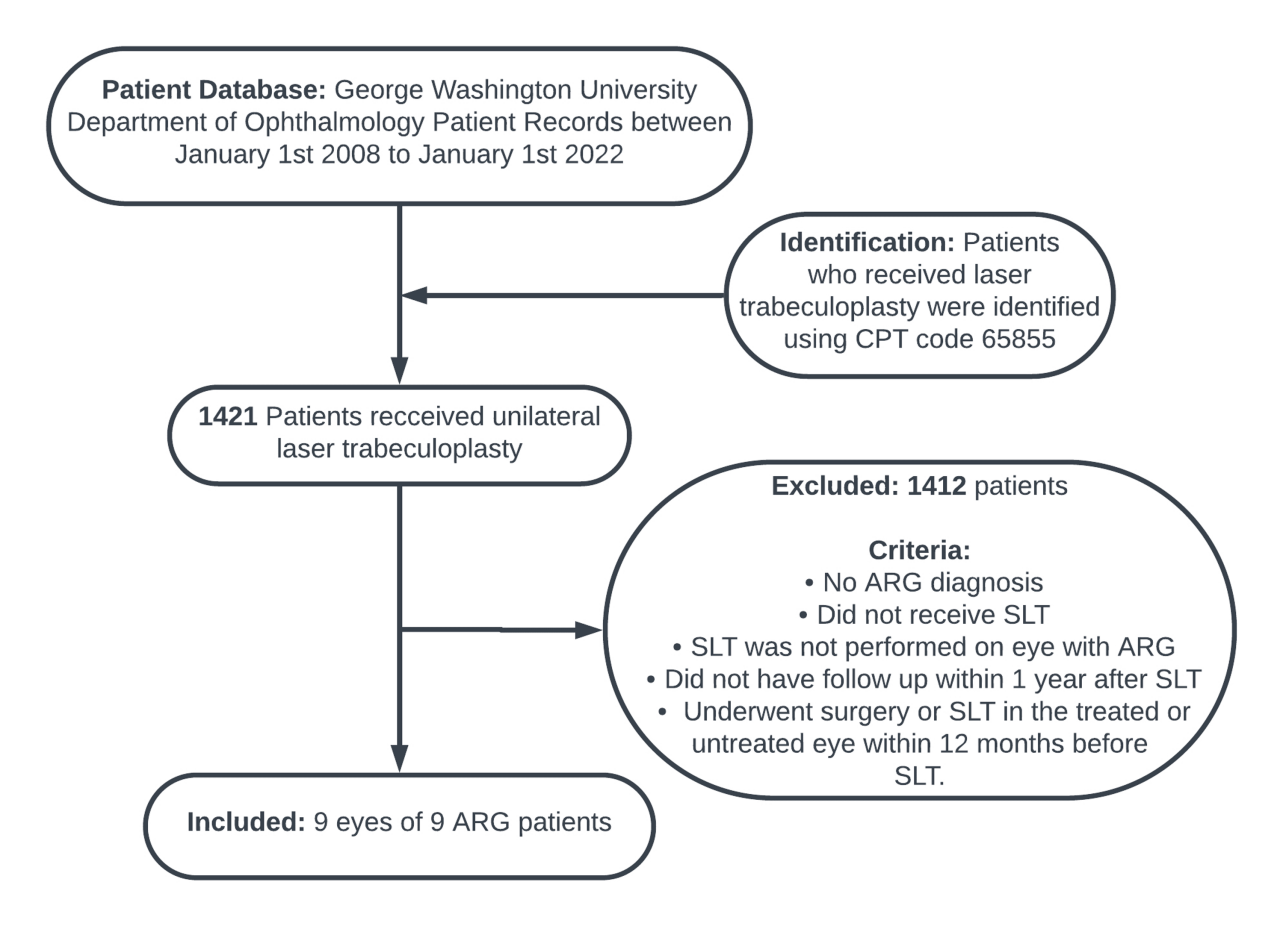
Inclusion and exclusion criteria. CPT: Current Procedural Terminology; ARG: angle recession glaucoma; SLT: selective laser trabeculoplasty.

This retrospective observational study examined charts of patients visiting the George Washington University (GWU) Department of Ophthalmology from January 1, 2008, to January 1, 2022, who underwent SLT. Inclusion and exclusion criteria are illustrated in Figure 1. Institutional Review Board approval was obtained from the George Washington University School of Medicine and Health Sciences (IRB number: NCR224001), and the study was exempt from informed consent because of its retrospective nature. All procedures and data collection were performed in accordance with compliance guidelines outlined by the Health Insurance Portability and Accountability Act and the tenets of the Declaration of Helsinki.

SLT procedure details

Four ophthalmologists at the GWU Department of Ophthalmology performed SLT in this case series. SLT was performed as detailed by Latina and colleagues [13]. After obtaining patient consent, topical tetracaine (0.5%) and proparacaine (0.5%) drops were instilled, followed by Iopidine before the procedure. SLT settings utilized a Q-switched, frequency-doubled Nd:YAG 532 nm laser with a 3-ns pulse and 400 μm spot size. Laser application ranged from 180 to 360 degrees, avoiding peripheral anterior synechiae and targeting visible areas of trabecular meshwork (TM). Energy settings varied from 0.5 to 1.2 millijoules.

Statistical analysis

Our primary outcome was treatment success, defined as a reduction in IOP of ≥20% from baseline after SLT, achieved without the need for additional medications or IOP-lowering procedures. This success criterion for SLT treatment was first described by Melamed et al. and was assessed at all follow-up visits (six weeks, six months, and 12 months) [14]. Variables included patient age, sex, race, history of trauma, years between trauma to the diagnosis of ARG, years between ARG diagnosis and SLT treatment, degrees of angle recession, baseline best corrected visual acuity (BCVA), IOP, number of medications, cup-to-disc (C/D) ratio, lens status, history of glaucoma surgery, and the diagnosis and severity in the untreated eye. Continuous variables such as IOP were compared across time points (baseline, six weeks, six months, and 12 months) using paired t-tests to assess the within-subject changes. For comparisons across multiple time points, we applied a one-way analysis of variance to evaluate differences in mean IOP and medication usage over the three follow-up periods. The Wilcoxon rank-sum test was used to perform non-parametric comparisons. All analyses were performed using statistical software R version 4.2.1 (R Foundation for Statistical Computing, Vienna, Austria) with a significance level set at p < 0.05.

## Results

A total of nine eyes of nine ARG patients who received SLT were included in the study (66.7% African American patients, 22.2% Caucasian patients, and 11.1% Hispanic/Latino patients). The mean age was 62.33 ± 17.32 years (range = 28-86 years), with five females (55.6%) and four males (44.4%). Table 1 presents the baseline demographic and clinical variables for each patient and Table 2 details the laser settings. Notably, cases 2 and 3 refer to the same patient, who underwent two separate SLT treatments three years apart. These cases are presented individually to highlight longitudinal outcomes.

**Table 1:** Baseline demographics and clinical characteristics of treated eyes. ARG = angle recession glaucoma; POAG = primary open-angle glaucoma; SLT = selective laser trabeculoplasty; BCVA = best corrected visual acuity; IOP = intraocular pressure; Meds = medications; C/D = cup-to-disk; Hx = history; HM = hand motion; CF = counting fingers.

Patient	Age	Sex	Race	Hx of trauma?	Trauma to ARG (years)	ARG to SLT (years)	Degrees of angle recession	Baseline BCVA	Baseline IOP (mmHg)	Baseline meds (#)	C/D ratio	Lens status	Hx of glaucoma surgery?	Untreated eye diagnosis and severity
Case 1	49	Male	Black	Yes	32	2	270	HM	22	3	1.0	Phakic	No	POAG mild
Case 2	60	Female	Black	Yes	35	1	360	CF at 5 feet	20	4	1.0	Pseudophakic	Yes	POAG suspect
Case 3	63	Female	Black	Yes	38	3	360	CF at face	30	4	1.0	Pseudophakic	Yes	POAG suspect
Case 4	28	Female	Hispanic/Latino	Yes	7	0.5	180	20/20	14	1	0.6	Phakic	No	POAG suspect
Case 5	73	Male	White	Yes	36	6	360	20/150	17	1	0.8	Phakic	No	None
Case 6	86	Male	White	No	0	10	270	20/30	17	4	0.95	Pseudophakic	No	POAG suspect
Case 7	77	Male	Black	No	0	10	60	20/40	12	4	0.55	Phakic	No	POAG suspect
Case 8	71	Male	Black	Yes	5	1	90	20/40	29	3	0.3	Pseudophakic	No	None
Case 9	55	Female	Black	Yes	39	0.5	180	20/40	20	4	0.95	Phakic	No	POAG suspect

**Table 2:** SLT settings for ARG eyes and follow-up complications. SLT = selective laser trabeculoplasty; ARG = angle recession glaucoma; IOP: intraocular pressure.

Patient	Degrees of treatment	Power per shot (mJ/shot)	Spot count	Total energy (mJ)	Post-SLT IOP spike?	Post-SLT uveitis?
Case 1	360	1.0	100	122	No	No
Case 2	360	1.0	111	111	No	No
Case 3	360	1.0	111	111	No	No
Case 4	180	1.2	91	109	No	No
Case 5	360	1.0	151	173	Yes	No
Case 6	360	1.2	150	180	No	No
Case 7	360	0.9	113	101	Yes	No
Case 8	180	0.5	51	25.5	No	No
Case 9	180	1.0	52	52	No	No

Treatment outcomes

Treatment success in the ipsilateral eye was observed in five out of eight patients (62.5%) at various time points (Table 3). In the contralateral eyes, success was achieved in three out of eight patients (37.5%) (Table 4). BCVA remained stable or showed minimal changes in most patients.

**Table 3:** One-year follow-up outcomes in the treated ARG eye post SLT. ARG = angle recession glaucoma; SLT = selective laser trabeculoplasty; IOP = intraocular pressure; BCVA = best corrected visual acuity; Pre-Op = preoperative; Wk = weeks; Mo = months; HM = hand motion; CF = counting fingers.

Patient	Success timepoints	IOP				Number of medications				BCVA			
		Pre-Op	6 Wk	6 Mo	12 Mo	Pre-Op	6 Wk	6 Mo	12 Mo	Pre-Op	6 Wk	6 Mo	12 Mo
Case 1	6 Wk, 6 Mo, 12 Mo	22	15	16	16	3	3	3	3	HM	HM	HM	HM
Case 2	Failure	20	17	20	17	4	4	4	4	CF at 5 feet	CF at 5 feet	CF at 5 feet	CF at 5 feet
Case 3	6 Wk, 6 Mo, 12 Mo	30	20	21	23	4	4	4	4	CF at face	CF at face	CF at face	CF at face
Case 4	6 Mo	14	14	9.5	-	1	1	0	-	20/150	20/150	20/150	-
Case 5	Failure	16	17	-	-	1	2	-	-	20/30	20/30	-	-
Case 6	6 Wk, 6 Mo, 12 Mo	17	9	9	12	4	4	4	4	20/40	20/40	20/25	20/40
Case 7	Failure	12	18	20	19	4	3	3	3	20/40	20/40	20/25	20/40
Case 8	6 Mo, 12 Mo	29	28	22	14	3	3	3	3	20/40	20/80	20/50	20/60
Case 9	Failure	20	19	26	-	4	4	4	-	20/40	20/40	20/70	-

**Table 4:** One-year follow-up outcomes in the untreated eye post SLT. SLT = selective laser trabeculoplasty; IOP = intraocular pressure; Pre-Op = preoperative; Wk = weeks; Mo = months.

Patient	Success timepoints	IOP				Number of medications			
		Pre-Op	6 Wk	6 Mo	12 Mo	Pre-Op	6 Wk	6 Mo	12 Mo
Case 1	6 Wk	20	15	17	21	1	1	1	1
Case 2	Failure	18	18	20	17	0	0	0	0
Case 3	Failure	18	16	16	18	0	0	0	0
Case 4	Failure	12	14	14	-	0	0	0	-
Case 5	Failure	16	17	-	-	1	1	-	-
Case 6	6 Mo	11	9	6	12	2	2	2	2
Case 7	Failure	11	13	14	12	1	1	1	1
Case 8	Failure	14	15	15	12	0	0	0	0
Case 9	6 Wk, 6 Mo	15	9	11	-	4	4	4	-

**Figure 2:**
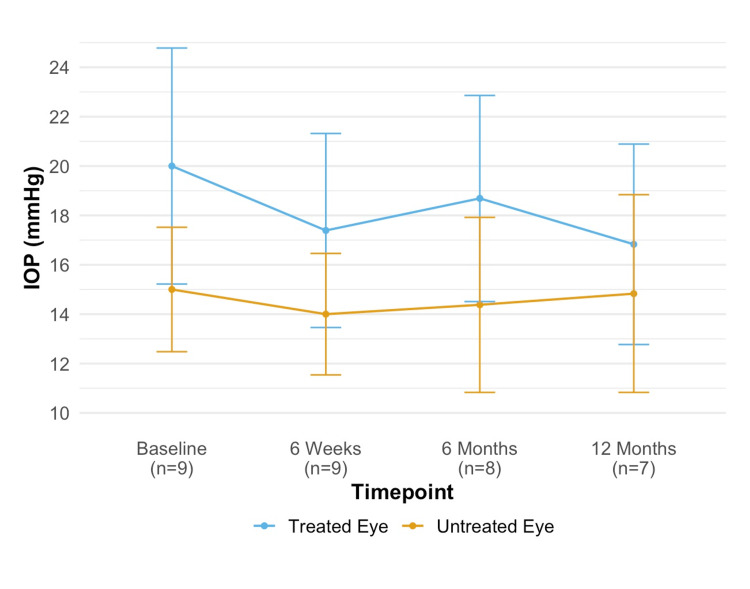
IOP at baseline and post SLT. IOP = intraocular pressure; SLT = selective laser trabeculoplasty.

The mean IOP for SLT-treated eyes at baseline, six-week, six-month, and 12-month visits was 20 ± 6.22 mmHg, 17.39 ± 5.11 mmHg (P = 0.36), 18.69 ± 4.99 mmHg (P = 0.49), and 16.83 ± 3.87 mmHg (P = 0.18), respectively. The p-values reflect comparisons between the baseline and each subsequent time point. For the same time points, the mean IOP for untreated eyes was 15 ± 3.28 mmHg, 14 ± 3.20 mmHg (P = 0.52), 14.38 ± 4.24 mmHg (P = 0.61), and 14.83 ± 3.82 mmHg (P = 0.83), respectively. IOP changes over time for each eye are illustrated in Figure 2.

**Figure 3:**
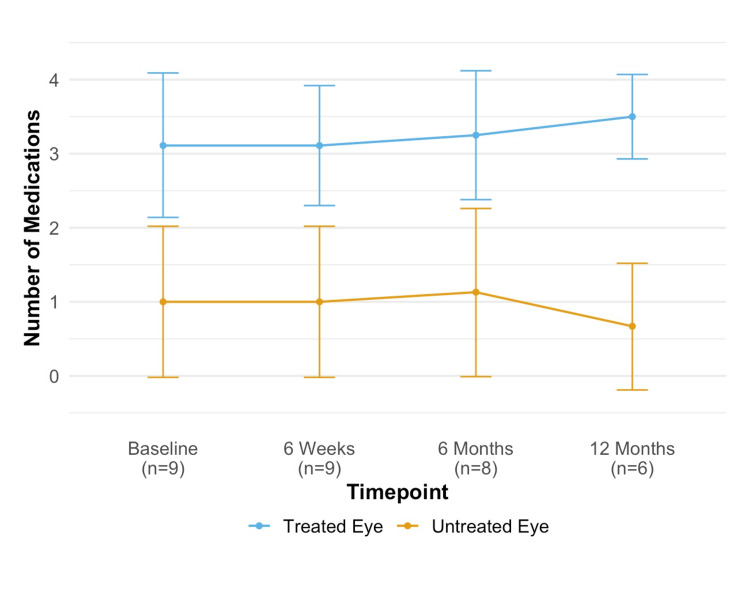
Number of medications at baseline and post SLT. SLT = selective laser trabeculoplasty.

The average number of medications used for the ipsilateral eye at baseline, six-week, six-month, and 12-month visits was 3.11 ± 1.27, 3.11 ± 1.05 (P = 1.0), 3.25 ± 1.04 (P = 0.81), and 3.50 ± 0.55 (P = 0.60), with no significant changes over time (P > 0.05). For the contralateral eye, the average medication use at those same points was 1.00 ± 1.32, 1.00 ± 1.32 (P = 1.0), 1.13 ± 1.36 (P = 1.0), and 0.67 ± 0.82 (P = 1.0), also showing no significant changes. Changes in medication use are illustrated in Figure 3.

Case series

Case 1

A 49-year-old male presented to the clinic with blurry near vision and a history of left eye trauma from a ball bearing (BB) gun 32 years prior. Baseline BCVA was 20/60 OS (left eye), and IOP was 57 mmHg OS. Fundus examination revealed a C/D ratio of 1.0 OS. Gonioscopy showed angle recession in three quadrants. The patient was started on Xalatan OU (both eyes) and Cosopt OS. Due to medication compliance and follow-up issues, IOP rose to 32 mmHg OS, and BCVA declined to hand motion over two years. SLT was performed with a preoperative IOP of 22 mmHg OS. Post SLT, IOP remained controlled between 15 and 16 mmHg OS over 12 months, with stable BCVA.

Cases 2 and 3

A 60-year-old female with a history of surgical trauma to the right eye during cataract extraction and a failed trabeculectomy presented to the clinic for evaluation. Baseline BCVA was counting fingers at 5 feet OD (right eye), and IOP was 24 mmHg OD. Gonioscopy indicated angle recession in all quadrants. The patient was on multiple glaucoma medications. SLT was first performed one year post ARG diagnosis (case 2) with a preoperative IOP of 20 mmHg OD. IOP remained between 17 and 20 mmHg OD over 12 months post SLT. Three years later, after being lost to follow-up, the patient returned with elevated IOP (case 3). A repeat SLT was performed with a preoperative IOP of 30 mmHg OD. Post-repeat SLT, IOP ranged from 20 to 23 mmHg OD over 12 months, but the patient declined further surgical intervention.

Case 4

A 28-year-old female with a traumatic eyebrow injury presented to the clinic with BCVA of 20/20 OD and IOP of 12 mmHg OD. Fundus examination revealed a C/D ratio of 0.6 OD. Gonioscopy showed angle recession in two quadrants. Due to medication compliance issues, SLT was performed four months after ARG diagnosis with a preoperative IOP of 14 mmHg OD. Post SLT, IOP decreased to 9.5-14 mmHg OD over six months, and the patient was able to discontinue medications but was lost to follow-up thereafter.

Case 5

A 73-year-old male with a history of right eye trauma from a champagne cork 36 years prior presented to the clinic with BCVA of 20/150 OD and IOP of 17 mmHg OD. Fundus examination showed a C/D ratio of 0.8 OD. Despite maximally tolerated medications, IOP remained elevated. SLT was performed with a preoperative IOP of 17 mmHg OD. Post SLT, IOP remained at 16-17 mmHg OD. Due to insufficient IOP control, an Ahmed glaucoma valve was implanted before the six-month follow-up.

Case 6

An 86-year-old male presented to the clinic without a history of eye trauma but with a history of extracapsular cataract extraction with posterior chamber intraocular lens implantation OS 20 years prior, and a 10-year history of ARG OS and primary open-angle glaucoma (POAG) suspect OD. His baseline BCVA was 20/30 OS, and his IOP was 17 mmHg OS on four medications. A gonioscopy of the left eye revealed angle recession in three quadrants. SLT was performed due to elevated IOP and visual field progression. Post SLT, his IOP decreased to 9-12 mmHg OS over 12 months, with stable visual fields.

Case 7

A 77-year-old male, without a history of eye trauma, presented to the clinic with a 10-year history of ARG OS. His baseline BCVA was 20/40 OS, and his IOP was 18 mmHg OS. Gonioscopy indicated superior angle recession over two clock hours OS. Due to an allergic reaction to medications and insufficient IOP control, SLT was performed with a preoperative IOP of 12 mmHg OS. Post SLT, IOP ranged from 18 to 20 mmHg OS over 12 months, and medications were adjusted accordingly.

Case 8

A 71-year-old male with non-proliferative diabetic retinopathy and a history of complicated cataract extraction presented to the clinic with BCVA of 20/40 OS and IOP of 29 mmHg OS. Gonioscopy indicated angle recession in one quadrant, inferiorly. SLT was performed with a preoperative IOP of 29 mmHg OS. Post SLT, IOP decreased to 14-28 mmHg OS over 12 months on the same medications.

Case 9

A 55-year-old female with a history of right eye trauma from a softball and bilateral SLT presented to the clinic with BCVA of 20/40 OD and IOP of 30 mmHg OD after discontinuing medications for three years due to lack of insurance. A gonioscopy in the right eye revealed angle recession in two quadrants superiorly. Repeat SLT was performed with a preoperative IOP of 20 mmHg OD. Post SLT, IOP ranged from 19 to 26 mmHg OD over six months, but the patient was lost to follow-up after agreeing to surgical intervention.

## Discussion

This study evaluated the effects of SLT on IOP and medication use in both treated ARG eyes and untreated contralateral eyes. While average IOP reductions at six weeks, six months, and 12 months were not statistically significant, we found that SLT achieved meaningful IOP control in certain individual ARG patients. Successful responses showed effective IOP reduction lasting up to 12 months in treated eyes and up to six months in untreated eyes.

Our cohort experienced SLT treatment success in five out of nine cases (55.5%) in eyes with ARG. This success rate, while comparable to the success rate of three out of four cases (75%) reported by AlObaida et al., has several key differences [9]. Notably, our study included a larger and more diverse patient population with a broader range of baseline IOPs, which could have influenced treatment outcomes. For instance, despite the pre-existing surgical status of case 3 (trabeculectomy), a successful outcome was still achieved. This could be attributed to a high baseline IOP, which has been shown to be a predictor of SLT success in eyes with OAG [12,15-17]. Furthermore, cases that experienced success generally had higher IOP than those that failed (Table 3). Moreover, our study involved eyes with varying degrees of angle recession and treatment, with some patients showing full 360-degree involvement and treatment, while others had partial recession ranging from 90 to 270 degrees, with 180 to 360 degrees of treatment (Table 2). In contrast, AlObaida et al. treated cases with only 90 to 180 degrees of angle recession [9]. Our findings suggest that SLT may be effective across a broader range of angle recession severity. Targeting partially recessed or damaged portions of the trabecular meshwork can potentially stimulate aqueous outflow and reduce IOP effectively. Additionally, the success observed may be due to treating 180 degrees of trabecular meshwork or more, as greater degrees of treatment have been shown to result in increased IOP lowering [18,19]. However, failures were observed in cases with 60 to 360 degrees of recession, despite receiving 180 to 360 degrees of treatment, highlighting the difficulty in treating ARG and predicting treatment outcomes.

Success in the untreated eye was observed in three out of nine patients (33.3%). This rate is comparable to findings from other studies investigating the contralateral effect of SLT, which reported a similar success rate of 36.8% [20]. To our knowledge, this is the first report documenting treatment success in the contralateral eye of patients with ARG. A possible mechanism behind this effect may involve not only local but also systemic activation of cytokines by SLT, which could enhance aqueous outflow in the contralateral eye [12]. Although the contralateral eyes that benefited had a history of suspected POAG and may have experienced a secondary benefit from SLT, the reduction is clinically insignificant and does not warrant a change in glaucoma management. Additionally, our study adds to the existing literature on ARG by documenting an increased prevalence of contralateral POAG or POAG suspect in this population. Previous research by Tesluk and Spaeth indicated that patients with ARG have a 50% likelihood of developing open-angle glaucoma in the non-ARG eye [7]. In our case series of nine ARG patients, six untreated eyes were diagnosed as POAG suspects, one eye had POAG, and two eyes were normal. Since our study did not determine whether the POAG suspect or POAG diagnosis occurred before or after the ARG diagnosis, we can only report an increased prevalence, not incidence, of contralateral POAG in ARG patients.

Previous studies have reported IOP spikes, defined as an increase of more than 5 mmHg from baseline within one hour post SLT, in eyes with POAG. In our study, two out of nine cases (25%) experienced an IOP spike. This is consistent with other studies, which have reported IOP spikes greater than 5 mmHg in up to 28% of eyes [21]. No other complications such as post-SLT uveitis were observed during follow-up, further supporting the safety of SLT in eyes with ARG.

Our study provides a unique perspective on the effectiveness and safety of SLT in treating ARG. While we did not find a statistically significant reduction in IOP overall, individual patients did benefit from SLT in the treated or untreated eye. However, our study has limitations: it was retrospective, had a small sample size with one repeat laser, spanned 14 years with multiple physicians performing the procedure, included two treatments from the same patient, and had patients lost at follow-up, affecting data analysis at the six-month and 12-month marks. The inclusion of multiple treatments from one patient may introduce bias, and future studies may benefit from limiting to one treatment per patient. While the involvement of different providers over an extended period may introduce some variability, it also enhances the generalizability of our findings by reflecting real-world practice. Larger, prospective studies are needed to confirm the safety and efficacy of SLT in eyes with ARG.

## Conclusions

SLT effectively reduced IOP in five out of nine (55.5%) treated eyes, although the overall reduction was not statistically significant. SLT also showed limited effectiveness in untreated contralateral eyes, with successful lowering of IOP in three out of nine (33%) untreated eyes. While the procedure was generally safe, individual responses varied, with greater success in cases with higher baseline IOP. These findings suggest that SLT may be a viable treatment option for patients with ARG, especially those seeking to delay more invasive surgeries. However, the variability in outcomes emphasizes the need for larger prospective studies to further evaluate the long-term efficacy and identify predictors of success in this population.
